# BIOMARKERS OF HEALTH AND AGING IN A COHORT OF VIETNAMESE ADULTS AGED 60 AND OLDER

**DOI:** 10.1093/geroni/igae098.4133

**Published:** 2024-12-31

**Authors:** Robert Tennyson, Melanie Martin, Tiffany Pan, Kim Korinek, Zachary Zimmer, Alan A Cohen, Toan Tran

**Affiliations:** Vanderbilt University, Nashville, Tennessee, United States; University of Washington, Seattle, Washington, United States; University of Washington, Seattle, Washington, United States; University of Utah, Salt Lake City, Utah, United States; Mount Saint Vincent University, Halifax, Nova Scotia, Canada; Columbia university, New-York, New York, United States; Hanoi Medical University, Hanoi, Ha Noi, Vietnam

## Abstract

War and traumatic events have negative health impacts across the life course, and most wars are situated in lower- and middle-income countries, where many survivors are now or will soon be entering old age. To date, however, most aging research has focused on health effects in high-income countries, neglecting the burdens of war exposure for the broader global population. The Vietnam Health and Aging Study (VHAS), a longitudinal study of 2,447 individuals aged 60+, investigates how stress and trauma experienced during the American War impacts health and aging of Vietnamese survivors. Using biomarker and anthropometric data collected in 2021-2022, we examined associations of recruitment characteristics (age, sex, district, military participation) with select cardiometabolic and inflammatory biomarkers (e.g., C-reactive protein [CRP], Interleukin-6 [IL-6]). Preliminary analyses revealed variation in associations of key demographic and outcome measures. Hypertensive risks were significantly higher for males, and overweight/obesity (OW/OB) risk significantly higher for females, whereas age was positively associated with hypertensive risk and inversely associated with OW/OB risk. Hypertensive risk was lower for those with formal military experience, while proinflammatory markers (i.e., CRP, IL-6) increased with age but did not vary by sex or military participation. District residence, which reflects differences in bombing intensity during the war, showed varied associations with inflammatory and cardiometabolic biomarkers. These findings suggest inflammatory and cardiometabolic risks are not uniformly coupled across participants in this study, underscoring the complexities of how prior war trauma interacts with other factors to influence health and well-being during aging.

